# Aberrant expression and potency as a cancer immunotherapy target of alpha-methylacyl-coenzyme A racemase in prostate cancer

**DOI:** 10.1186/1479-5876-7-103

**Published:** 2009-12-09

**Authors:** Ichiya Honma, Toshihiko Torigoe, Yoshihiko Hirohashi, Hiroshi Kitamura, Eiji Sato, Naoya Masumori, Yasuaki Tamura, Taiji Tsukamoto, Noriyuki Sato

**Affiliations:** 1 Department of Pathology, Sapporo Medical University School of Medicine, Sapporo, Japan; 2 Department of Urology, Sapporo Medical University School of Medicine, Sapporo, Japan

## Abstract

Alpha-methylacyl-CoA racemase (AMACR) is an enzyme playing an important role in the beta-oxidation of branched-chain fatty acids and fatty acid derivatives. High expression levels of AMACR have been described in various cancers, including prostate cancer, colorectal cancer and kidney cancer. Because of its cancer-specific and frequent expression, AMACR could be an attractive target for cytotoxic T-lymphocyte (CTL)-based immunotherapy for cancer. In the present study, we examined the induction of AMACR-specific CTLs from prostate cancer patients' peripheral blood mononuclear cells (PBMCs) and determined HLA-A24-restricted CTL epitopes.

RT-PCR and immunohistochemical analysis revealed that AMACR was strongly expressed in prostate cancer cell lines and tissues as compared with benign or normal prostate tissues. Four AMACR-derived peptides carrying the HLA-A24-binding motif were synthesized from the amino acid sequence of this protein and analyzed to determine their binding affinities to HLA-A24. By stimulating patient's PBMCs with the peptides, specific CTLs were successfully induced in 6 of 11 patients. The peptide-specific CTLs exerted significant cytotoxic activity against AMACR-expressing prostate cancer cells in the context of HLA-A24. Our study demonstrates that AMACR could become a target antigen for prostate cancer immunotherapy, and that the AMACR-derived peptides might be good peptide vaccine candidates for HLA-A24-positive AMACR-expressing cancer patients.

## Introduction

Cytotoxic T lymphocytes (CTLs) play a major role in the anti-cancer immune response [1]. Thus far, large numbers of tumor-associated antigens and their CTL epitopes have been identified [2,3]. High-throughput gene expression profiling using a cDNA microarray allows for systematic interrogation of transcriptionally altered genes. By comparing the mRNA expression profiles of cancerous lesions with non-cancerous lesions, a number of candidate antigens for tumor-specific immunotherapy have emerged. CTL epitope peptides derived from tumor-specific antigens like the MAGE gene family have been employed for pioneering studies of immunotherapy in cases of advanced melanoma patients [4,5].

Castration-resistant prostate cancer is an aggressive disease with limited treatment options. Hence, there is great need for new therapeutic strategies to treat prostate cancer, and recent progress in understanding of tumor immunology has raised expectations that antigen-specific immunotherapy may become a new modality for cancer therapy. Alpha-methylacyl coenzyme A racemase (AMACR) was identified as one of the genes that were highly expressed in prostate cancer tissues through gene expression profiling using a DNA microarray and RT-PCR [6-8]. AMACR is an enzyme that catalyzes the racemization of alpha-methyl carboxylic coenzyme A thioesters in mitochondria and peroxisomes [9,10]. AMACR is expressed abundantly in prostate cancer tissues as well as colorectal cancer and lung cancer tissues, whereas it is barely detected in benign tissues and normal prostate epithelial cells [6-8]. Immunohistochemical staining for AMACR is currently used in the clinical setting to support the histological diagnosis of prostate cancer. Because it has characteristics of cancer-specific expression and frequent expression in various cancers, AMACR is an attractive target for cancer immunotherapy. In the present study, we examined the induction of AMACR-specific CTLs from prostate cancer patients' peripheral blood mononuclear cells (PBMCs) and determined HLA-A24-restricted CTL epitopes. Our study demonstrates for the first time HLA-A24-restricted AMACR-derived CTL epitopes that might be suitable for peptide vaccines for AMACR-expressing cancer patients.

## Materials and methods

### Tissue Samples and PBMC

Surgically resected tissue specimens and PBMCs were obtained from HLA-A*2402-positive prostate cancer patients who were treated at Sapporo Medical University Hospital (Sapporo, Japan) after obtaining their informed consent. The study was approved by the Institutional Review Board for Clinical Research at our university. The expression of HLA-A24 molecules on PBMCs of cancer patients was determined by flow cytometry using an anti-HLA-A24 monoclonal antibody (c7709A2.6, kindly provided by Dr. P. G. Coulie, Ludwig Institute for Cancer Research, Brussels Branch).

### Cell Lines and Culture

Prostate cancer cell lines (LNCaP, DU145, and PC-3) and proerythroleukemia cell line K562 were cultured in RPMI 1640 (Sigma, St. Louis, MO) supplemented with 10% fetal bovine serum (FBS) (Filtron, Brooklyn, Australia). T2-A*2402 cells, which are transporters associated with antigen processing (TAP)-deficient T2 cells transfected with HLA-A*2402 complementary DNA (cDNA) were cultured in RPMI 1640 supplemented with 10% fetal bovine serum and 800 μg/mL G418 (Invitrogen Life Technologies Co., Carlsbad, CA). LNCaP and DU145 are HLA-A*2402-negative prostate cancer cell lines. To generate LNCaP and DU-145 sublines expressing HLA-A24, HLA-A*2402 cDNA was transduced into the cells by electroporation using a Gene Pulser (Bio-Rad, Richmond, CA) as reported previously [11]. The expression of HLA-A24 molecules on the cell lines was determined by flow cytometry using the anti-HLA-A24 monoclonal antibody. LNCaP-A*2402 and DU145-A*2402, stable HLA-A*2402 transfectants of LNCaP and DU145 cells, respectively, were established and cultured in RPMI 1640 supplemented with 10% FBS and 500 ng/ml puromycin (Sigma).

### Reverse transcriptase-polymerase chain reaction (RT-PCR)

Multiple Tissue cDNA Panels (BD Biosciences Clontech, Palo Alto, CA) were used as a template of normal tissue cDNA. Total RNA was extracted using an RNeasy kit (Qiagen, Hilden, Germany). A cDNA mixture was synthesized from 1 μg of total RNA by reverse transcription (RT) using Superscript II and oligo (dT) primer (Invitrogen Life Technologies) according to the manufacturer's protocol. PCR amplification was done in 50 μL of PCR mixture containing 1 μL of the cDNA mixture, 1 μL of KOD Plus DNA polymerase (TOYOBO, Osaka, Japan) and 15 pmol of primers. For specific detection of AMACR, forward primer 5'-CGG GGT ACC ATG GCA CTG CAG GGC ATC TCG-3' and reverse primer 5'-ATA AGA ATG CGG CCG CGA GAC TAG CTT TTA CCT TAT TAC T-3' were employed. As an internal control, β-actin expression was detected by using forward primer 5'-ACT GGC TCG TGA TGG ACT C-3' and reverse primer 5'-TCA GGC AGC TCG TAG CTC TT-3'. The amplification protocol consisted of denaturation for 15 seconds at 98°C, annealing for 45 seconds at 58°C and extension for 4 minutes at 72°C for a total of 30 cycles, using a GeneAmp PCR system model 2400 (Perkin-Elmer, Foster City, CA).

### Immunohistochemical Staining of Tissue Sections

Immunohistochemical staining was done with formalin-fixed paraffin-embedded tissue sections of surgically resected prostate cancer specimens. Four- to 5-μm-thick sections were deparaffinized in xylene and rehydrated in graded alcohols. Antigen retrieval was done by boiling sections for 20 minutes in a microwave oven in preheated 0.01 mol/L sodium citrate buffer (pH 6.0). Endogenous peroxidase activity was blocked by 3% hydrogen peroxide in ethanol for 10 minutes. After blocking with 1% non-fat dry milk in phosphate-buffered saline (PBS) (pH 7.4), the sections were reacted with a rabbit polyclonal antibody to AMACR (clone RP134, Diagnostic BioSystems Co., Pleasanton, CA, USA) at 25 μg/mL or preimmune sera for 1 hour, followed by incubation with biotinylated goat anti-rabbit IgG (Nichirei, Tokyo, Japan) for 30 minutes. Subsequently, the sections were stained with streptavidin-biotin complex (Nichirei), followed by incubation with 3,3-diaminobenzidine and counterstaining with hematoxylin. The same tissues were immunostained with an anti-prostate-specific antigen (PSA) polyclonal antibody (DAKO, Denmark).

### Peptides and Cytokines

AMACR-derived peptides were synthesized from the amino acid sequence of AMACR based on the HLA-A24-binding motifs. AMACR-derived peptides were provided by Dainippon Sumitomo Pharmaceutical Co. (Osaka, Japan). Two peptides were used as control peptides, Epstein-Barr virus (EBV) LMP2-derived peptide (TYGPVFMSL) and human immunodeficiency virus (HIV) env-derived peptide (RYLRDQQLLGI), which have been shown to become CTL epitopes in the context of HLA-A*2402 previously [12,13], and ovalbumin-derived SL-8 peptide (OVA257-264, SIINFEKL) was used as a negative control peptide. These peptides were synthesized and purchased from Sigma Genosys (Ishikari, Japan). The peptides were dissolved in DMSO at a concentration of 5 mg/mL and stored at -80°C. Human recombinant interleukin (IL)-2, IL-4 and granulocyte macrophage colony-stimulating factor (GM-CSF) were kind gifts from Takeda Pharmaceutical Co. (Osaka, Japan), Ono Pharmaceutical Co. (Osaka, Japan) and Novartis Pharmaceutical (Basel, Switzerland), respectively. Human recombinant IL-7 was purchased from Invitrogen Life Technologies.

### Peptide Binding Assay

Peptide binding affinity to the HLA-A24 molecule was assessed by HLA-A24 stabilization assay as described previously [13], based on the findings that MHC class I molecules could be stabilized on the cell surface in the presence of binding peptides. After incubation of T2-A*2402 cells in culture medium at 26°C for 18 hours, the cells (2 × 10^5^) were washed with PBS and suspended with 1 mL of Opti-MEM (Life Technologies) with or without 100 μg of peptide, followed by incubation at 26°C for 3 hours and then at 37°C for 3 hours. After washing with PBS, the cells were incubated with the anti-HLA-A24 monoclonal antibody at 4°C for 30 minutes, followed by incubation with fluorescein isothiocyanate (FITC)-conjugated rabbit anti-mouse IgG at 4°C for 30 minutes. The cells were then suspended with 1 mL of PBS containing 1% formaldehyde, and analyzed by FACScan (Becton Dickinson, Mountain View, CA). Binding affinity was evaluated by comparing mean fluorescence intensity (MFI) of HLA-A24 expression in the presence of peptide pulsation to MFI in the absence of the peptide.

### Peptide-specific CTL Induction with Immature Dendritic Cells and Phytohemagglutinin Blasts

PBMCs were isolated from prostate cancer patients by standard density gradient centrifugation on Lymphoprep (Nycomed, Oslo, Norway). PBMCs were incubated in AIM-V medium (Invitrogen Life Technologies, Inc.) supplemented with 2-mercaptoethanol (50 μM) and HEPES (10 mM) for 2 hours at 37°C in a culture flask to separate adherent cells and non-adherent cells. Adherent cells were then cultured in the presence of IL-4 (1000 units/ml) and GM-CSF (1000 units/ml) in AIM-V medium for 7 days to generate monocyte-derived dendritic cells (DCs). The adherent cells containing DCs and phytohemagglutinin (PHA)-stimulated blasts were used as antigen-presenting cells (APCs). CD8-positive T lymphocytes were isolated from non-adherent cells with the MACS separation system (Milteny Biotech, Bergish Blabach, Germany) using an anti-CD8 monoclonal antibody coupled with magnetic microbeads according to the manufacturer's instructions. To obtain PHA-stimulated blasts, CD8-negative non-adherent PBMCs were cultured in AIM-V medium containing 1 μg/ml PHA (WAKO Chemicals, Osaka, Japan) and 100 units/ml of IL-2 for 3 days, followed by washing and cultivation in the presence of IL-2 (100 units/ml) for 4 days.

CTLs were induced from PBMCs of cancer patients by using autologous DC and PHA-blasts as APCs as described previously [14,15]. Briefly, APCs were cultured in AIM-V medium supplemented with 50 μmol/L peptide at room temperature for 2 hours, followed by washing with AIM-V once, then irradiated (100 Gy) and used for stimulation of CTLs. The CTL induction procedure was initiated by stimulating CD8^+ ^cells with peptide-pulsed autologous DCs at a 20:1 effector/APC ratio in AIM-V supplemented with HEPES, 2-ME, and IL-7 (10 ng/mL) for 7 days at 37°C. The following stimulation was done with peptide-pulsed PHA-blasts at a 10:1 effector/APC ratio. On the day after the 2nd stimulation, IL-2 was added to the culture at a concentration of 10 units/mL. The same CTL stimulation cycle with PHA-blasts was then done twice more over a period of 2 weeks. One week after the 4th stimulation, cytotoxic activity of the CTL was measured by ^51^Cr release assay.

### Cytotoxicity Assay

The cytotoxic activities of CTLs were measured by ^51^Cr-release assay as described previously [16]. Target cells were labeled with 100 μCi of ^51^Cr for 1 hour at 37°C and washed with RPMI 1640 three times. Then ^51^Cr-labeled target cells were incubated with or without peptide and effector cells at various effector/target ratios at 37°C for 6 hours in V-bottomed 96-well microtiter plates. Then supernatants were collected and the radioactivity was measured with a gamma-counter. The % specific lysis was calculated as follows: % specific lysis = (test sample release - spontaneous release) × 100/(maximum release - spontaneous release). For peptide-pulsed target cells, T2-A*2402 cells were incubated with 1 μg/ml peptide at room temperature for 1 hour before the assay. Moreover, we also examined cytotoxic activity against LNCaP, LNCaP-A*2402, DU145 and DU145-A*2402 prostate cancer cells, which express endogenous AMACR.

### ELISPOT Assay

ELISPOT plates were coated sterilely overnight with an IFN-γ capture antibody (Beckton Dickinson Biosciences) at 4°C. The plates were then washed once and blocked with AIM-V medium containing 10% human serum for 2 hr at room temperature. CD8-positive T cells separated from patients' PBMCs (5 × 10^3 ^cells/well), which were stimulated in vitro with peptides, were then added to each well along with HLA-A24-transfected CIR cells (CIR-A24) (5 × 10^4 ^cells/well), which had been preincubated with the AMACR peptide (10 μg/ml) or HIV peptide as a negative control. After incubation in a 5% CO_2 _humidified chamber at 37°C for 24 hours, the wells were washed vigorously five times with PBS and incubated with a biotinylated anti-human IFN-γ antibody and horseradish peroxidase-conjugated avidin. Spots were visualized and analyzed using KS ELISPOT (Carl Zeiss, Germany).

### Statistical Analysis

We tested the statistical significance of cytotoxic activity of CTLs induced with peptides using Student's t-test. A value of p < 0.05 was considered to indicate statistical significance.

## Results

### AMACR Expression in Normal Tissues, Prostate Cancer Cell Lines and Cancer Tissues

**Figure 1:**
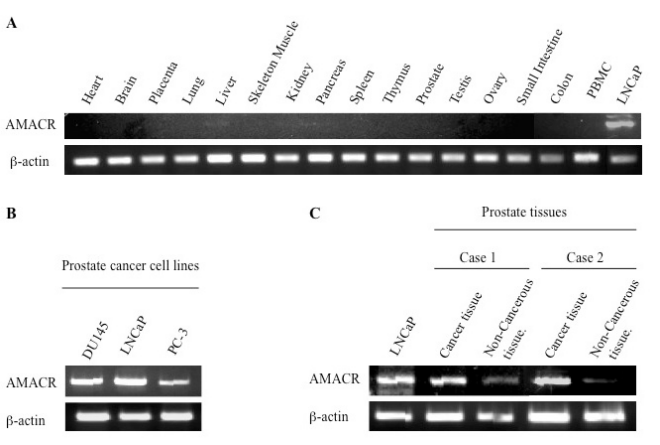
**Expression profiles of AMACR as assessed by RT-PCR**. A. Expression of AMACR in normal tissues including heart, brain, placenta, lung, liver, skeletal muscle, kidney, pancreas, spleen, thymus, prostate, testis, ovary, small intestine, colon and PBMC. LNCaP, a prostate cancer cell line was used as a positive control for AMACR expression. B. Expression of AMACR in prostate cancer cell lines. Beta-actin expression was detected as an internal control. AMACR mRNA was detected in three prostate cancer cell lines (LNCaP, DU145 and PC-3). C. Expression of AMACR in cancer tissues and noncancerous tissues from surgical specimens of two prostate cancer cases.

First the expression profile of AMACR in normal adult tissues by RT-PCR was difined. We detected the overt expression of β-actin mRNA and AMACR mRNA in prostate cancer line LNCaP, but only very weak expression of AMACR mRNA was observed in normal adult liver and pancreas (Figure 1A). In contrast, the AMACR mRNA level was elevated in all three prostate cancer cell lines (LNCaP, DU145 and PC-3) and in surgically resected prostate cancer tissues (Figure 1B and 1C). Low levels of expression were detected in non-cancerous prostate tissues (Figure 1C).

**Figure 2:**
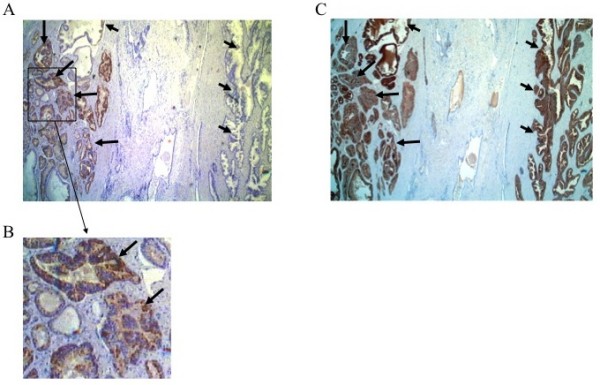
**Immunostaining of prostate cancer tissue with antibodies against AMACR and PSA**. Surgically resected prostate cancer tissue was immunostained with an anti-AMACR antibody (panel A) or anti-PSA antibody (panel C). The lower column (panel B) is a magnified view of the box of panel A. A clear distinction is noted between cancerous tissue with strongly positive AMACR staining (long arrow) and noncancerous glands without AMACR staining (short arrow) whereas both of them are positive for PSA.

Immunohistochemical analysis revealed that AMACR was present in prostate cancer tissues in 27 (69.2%) of the 39 patients (Figures 2A and 2B). AMACR was weakly detected in non-cancerous prostate tissues, but barely detected in normal essential tissues such as adult liver and pancreas by immunohistochemical staining. In contrast, PSA was stained in both prostate cancer tissue and non-cancerous tissue (Figure 2C). These data indicated that AMACR had a mostly cancer-specific expression profile at both the mRNA level and protein levels.

### AMACR-derived Peptides Carrying HLA-A24 Binding Motif

**Figure 3:**
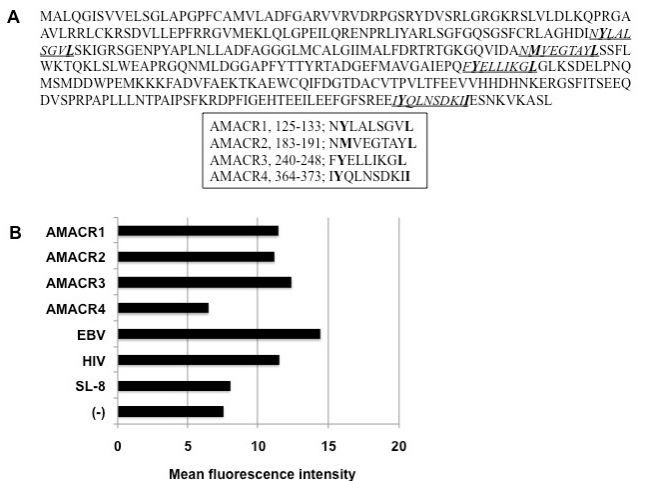
**Amino acid sequences of AMACR-derived peptides and their HLA-A24 binding assay**. A. Amino acid sequences of AMACR protein and four peptides (AMACR1-4) with HLA-A24 binding motif (underlines). The predicted anchor residues to HLA-A24 are indicated in boldface within the amino acid sequences of the peptides. B Binding affinities of AMACR-derived peptides to HLA-A24 molecule were evaluated by the mean fluorescence intensity (MFI) of cell surface HLA-A24 molecules on T2-A*2402 cells that were pulsed with each peptide. EBV LMP2-derived peptide (TYGPVFMSL) and HIV env-derived peptide (RYLRDQQLLGI) were used as positive controls for HLA-A24-bound peptides. SL-8 peptide (SIINFEKL) was used as a negative control.

Antigenic peptides derived from AMACR protein might be presented by HLA class I molecules and recognized by CD8-positive T cells. We focused on HLA-A*2402-restricted peptides because of its high frequency in Asian people. The amino acid sequence of AMACR protein was screened for peptides that had an HLA-A24 binding motif, such as 9- and 10-mer peptides with Y, F, M, or W at the 2nd position and L, I, F, or M at COOH-terminal position [17]. Consequently, we found four peptides, AMACR1 (NYLALSGVL), AMACR2 (NMVEGTAYL), AMACR3 (FYELLIKGL) and AMACR4 (IYQLNSDKII) carrying the HLA-A24 binding motif (Figure 3A). Next, we assessed their binding affinities to HLA-A24 molecules by a binding assay using TAP-deficient T2 cells transfected with HLA-A*2402. The MFI of cell surface HLA-A24 was clearly increased in the presence of positive control peptides, EBV peptide and HIV peptide, whereas it was not changed in the presence of negative control peptide SL-8, indicating the adequate qualification of this assay. The HLA-A24 level on the cell surface of T2-A*2402 cells was up-regulated in the presence of AMACR1, AMACR2 and AMACR3 peptides, but not in the presence of AMACR4 peptide, indicating that AMACR1, 2 and 3 peptides were possible HLA-A24-presentable peptides (Figure 3B).

### CTL Induction from PBMCs of HLA-A24-positive Prostate Cancer Patients

**Figure 4:**
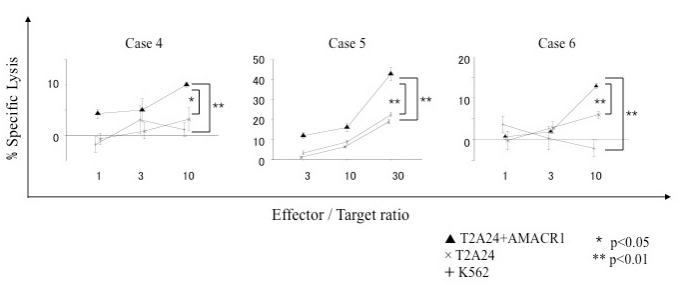
**AMACR1 peptide-specific CTL induction from PBMCs of HLA-A24-positive prostate cancer patients**. PBMCs of HLA-A24-positive prostate cancer patients (cases 4, 5 and 6) were stimulated four times with three kinds of AMACR peptide (AMACR1-3)-pulsed APCs and their cytotoxic activities were examined by ^51^Cr release assay at the indicated effector/target ratios. AMACR1 peptide-pulsed T2-A*2402 cells served as target cells. Non-pulsed T2-A*2402 cells were used as negative control target cells. K562 target cells were used for monitoring natural killer cell activity and lymphokine-activated nonspecific cytotoxicity.

**Figure 5:**
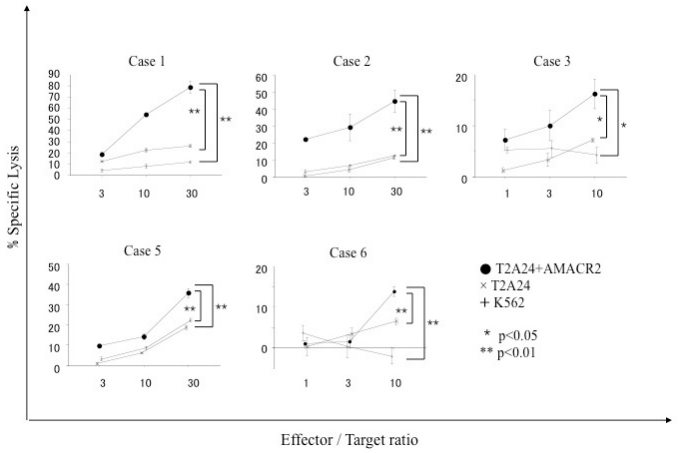
**AMACR2 peptide-specific CTL induction from PBMCs of HLA-A24-positive prostate cancer patients**. PBMCs of HLA-A24-positive prostate cancer patients (cases 1, 2, 3, 5 and 6) were stimulated four times with three kinds of AMACR peptide (AMACR1-3)-pulsed APCs and their cytotoxic activities were examined by ^51^Cr release assay at the indicated effector/target ratios. AMACR2 peptide-pulsed T2-A*2402 cells served as target cells. Non-pulsed T2-A*2402 cells and K562 cells were used as negative control target cells.

**Figure 6:**
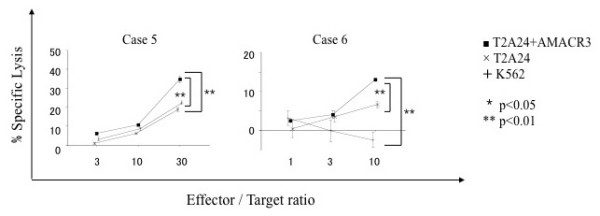
**AMACR3 peptide-specific CTL induction from PBMCs of HLA-A24-positive prostate cancer patients**. PBMCs of HLA-A24-positive prostate cancer patients (cases 5 and 6) were stimulated four times with three kinds of AMACR peptide (AMACR1-3)-pulsed APCs and their cytotoxic activities were examined by ^51^Cr release assay at the indicated effector/target ratios. AMACR3 peptide-pulsed T2-A*2402 cells served as target cells. Non-pulsed T2-A*2402 cells and K562 cells were used as negative control target cells.

**Figure 7:**
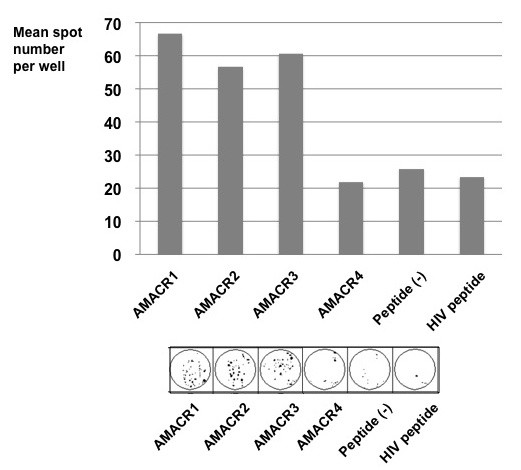
**Peptide-specific interferon-γ release of CTLs**. PBMCs of HLA-A24-positive prostate cancer patient (case 6) were stimulated four times with four kinds of AMACR peptide (AMACR1-4)-pulsed APCs and peptide-specific interferon-γ release was analyzed by ELISPOT assay. CTLs could release interferon-γ in response to AMACR1, 2 and 3 peptides, but not in response to AMACR4 peptide or HIV peptide.

We attempted to induce AMACR peptide-specific CTLs from PBMCs of HLA-A24 positive prostate cancer patients and assessed their cytotoxic activity. PBMCs were cultured with APCs pulsed with a mixture of three AMACR-derived peptides. After stimulation four times with the peptides, the cytotoxic activity against peptide-pulsed target cells was examined by ^51^Cr-release assay. The CTLs induced by the *in vitro *stimulation with AMACR peptides showed specific reactivity to the peptide-pulsed T2-A*2402 cells in 6 of 11 cases of HLA-A24-positive patients with AMACR-positive prostate cancer (Table 1 and Figures 4, 5, 6 and 7). CTLs could not be induced in any of the patients with AMACR-negative prostate cancer. In five cases (cases 1, 2, 3, 5 and 6) with AMACR-positive prostate cancer, CTLs reacting to AMACR2 peptide-pulsed T2-A*2402 cells were induced (Figure 5). With respect to AMACR1 and the 3 peptides, peptide-specific CTLs were induced in three cases (cases 4, 5 and 6, Figure 4) and two cases (cases 5 and 6, Figure 6), respectively. Since the cytotoxic activity of CTLs of case 6 was relatively low as compared with the other cases, peptide-specificity was assessed by ELISPOT assay. CTLs of case 6 could release interferon-γ in response to AMACR1, 2 and 3 peptides, but not in response to AMACR4 peptide or HIV peptide (Figure 7), indicating that the peptide specificity of the CTLs was consistent with the cytotoxic assay.

**Table 1:** Summary of clinicopathological characteristics and peptide-specific CTL induction from the peripheral blood mononuclear cells of prostate cancer patients

Case no.	Age (years old)	PSA (ng/ml)	Gleason score	Pathologic stage	AMACR expression	CTL induction	Peptide specificity
1	60	6.7	4+3	T2aN0M0	+	+	AMACR2
2	73	6.0	3+3	T2aN0M0	+	+	AMACR2
3	65	11.6	4+3	T2bN0M0	+	+	AMACR2
4	64	15.6	3+4	T3aN0M0	+	+	AMACR1
5	67	18.4	4+5	T3aN0M0	+	+	AMACR1,2,3
6	67	14.4	4+3	T2bN0M0	+	+	AMACR1,2,3
7	71	10.9	3+5	T3bN0M0	+	-	-
8	71	4.6	3+4	T2aN0M0	+	-	-
9	72	5.7	3+4	T2aN0M0	+	-	-
10	67	8.0	4+4	T2aN0M0	+	-	-
11	67	4.3	3+3	T2bN0M0	+	-	-
12	61	11.5	3+4	T2aN0M0	-	-	-
13	61	10.1	4+3	T2bN0M0	-	-	-
14	61	10.4	3+4	T2aN0M0	-	-	-
15	60	6.6	3+4	T2aN0M0	-	-	-

### Cytotoxic Activity of AMACR Peptide-specific CTLs Against HLA-A24-positive AMACR-positive Prostate Cancer Cell Lines

**Figure 8:**
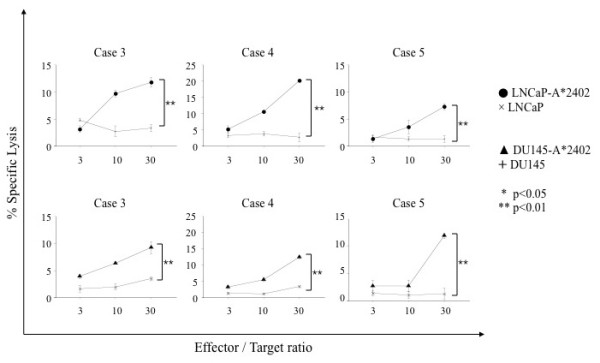
**Cytotoxic activities of AMACR peptide-specific CTLs against HLA-A24-positive AMACR-expressing prostate cancer cell lines**. AMACR peptide-specific CTLs were examined for the cytotoxic activity against HLA-A24-positive AMACR-expressing prostate cancer lines, LNCaP-A*2402 and DU145-A*2402, which were stable HLA-A*2402-transfectants of LNCaP and DU145, respectively. The cytotoxicity was assessed by ^51^Cr release assay at the indicated effector/target ratios.

To confirm that CTLs induced with AMACR peptides could exert cytotoxicity against AMACR-expressing prostate cancer cell lines in an HLA-A*2402-restricted manner, we examined their cytotoxic activity against prostate cancer cell lines that express endogenous AMACR by ^51^Cr-release assay. LNCaP-A*2402 and DU145-A*2402, which express both endogenous AMACR and gene-transfected HLA-A*2402, were used as target cells. Parental LNCaP and DU145 cells, HLA-A*2402-negative prostate cancer cells, were used as negative control target cells. As shown in Figure 8, CTLs induced from PBMCs of HLA-A*2402-positive prostate cancer patients (cases 3, 4 and 5) with AMACR peptides exerted cytotoxic activity against LNCaP-A*2402 and DU145-A*2402 cells but not against LNCaP and DU145 cells. These data implied that the peptide-specific CTLs were capable of recognizing endogenously processed AMACR-derived peptides in an HLA-A24-restricted manner.

## Discussion

Specific immunotherapy for cancer is anticipated to become an alternative or complementary therapy for recurrent or metastatic disease. Successful immunotherapy depends on the identification of cancer-specific antigens and the immunopotent CTL epitopes. Proteins that are selectively expressed in cancer cells, but not in normal adult tissues should become suitable targets for cancer-specific immunotherapy. To establish effective immunotherapy for prostate cancer, exploration of prostate cancer-specific antigens has been conducted.

Although prostate-specific antigen (PSA) is a well-known serum biomarker for prostate cancer, it has poor specificity to cancer. PSA is highly expressed in noncancerous prostatic tissues as well as in cancerous tissues [18-20] as shown in Figure 2B in the present study. Indeed, serum PSA levels are increased in patients with benign prostatic diseases such as benign prostatic hypertrophy and prostatitis. Recently, new prostate cancer antigens have been reported and examined as target antigens for cancer-specific immunotherapy [21-23]. In the present study, we focused on AMACR, a novel antigen that is overexpressed in a variety of tumor tissues, including prostate cancer.

AMACR was identified as a tissue biomarker for prostate cancer by gene expression profiling of primary human prostate cancer and benign prostatic hyperplasia (BPH) using cDNA microarrays [8]. Initial studies reported that AMACR was overexpressed in 94-100% of prostate cancers [6-8] though recent studies have demonstrated a slightly lower expression rate in the range of 80-90% for prostate cancer [24-26]. In our study, AMACR was detected in about 70% of prostate cancer cases by immunohistochemical analysis. This frequency was slightly lower than those of previous reports. On the other hand, its expression was very low in benign prostate glands, which showed only focal and weak staining [6]. The function of AMACR in prostate cancer has not been clarified yet. It has been reported that the function and expression of AMACR might be independent of androgen receptor signaling [27]. Recently, it has been reported that AMACR is overexpressed in various tumor tissues, including renal cell cancer, hepatic cancer, colon cancer and lung cancer. Because of the cancer specificity and high frequency of AMACR expression, it can be an attractive target for cancer immunotherapy. In this study, the immunogenic potency of AMACR-derived peptides was assessed using PBMCs from prostate cancer patients.

We focused on AMACR-derived peptides carrying the HLA-A24 binding motif. The HLA-A*2402 genotype is predominant in Japanese, accounting for about 60% of the population [28]. Four AMACR-derived peptides (AMACR1-4) carrying the HLA-A24-binding motif were identified in the present study. By stimulating peripheral blood lymphocytes of HLA-A24-positive/AMACR-expressing prostate cancer patients with these AMACR-derived peptides *in vitro*, peptide-specific CTLs were successfully induced in 4 of 9 patients. Moreover, the CTLs exerted significant cytotoxic activity against AMACR-expressing prostate cancer cells in the context of HLA-A24, indicating that AMACR-derived peptides might be useful as prostate cancer vaccines for HLA-A24-positive/AMACR-expressing prostate cancer patients. We demonstrated HLA-A24-restricted CTL responses against AMACR-derived peptides for the first time. Interestingly, the immunogenic peptides were distinct among the patients. However, it is likely that the AMACR2 peptide was the most immunogenic of the three AMACR-derived peptides.

There may be some problems in introducing new CTL-based immunotherapy for advanced recurrent and/or metastatic prostate cancer patients. Even after four rounds of *in vitro *stimulation of PBMCs with the peptides, cytotoxicity against AMACR-expressing tumor cells (% lysis) was only around 20% at a 30:1 E:T ratio. Such weak cytotoxicity may be insufficient to induce a clinical anti-tumor response. Since AMACR is involved in the bile acid synthesis and there is weak expression in the liver, it is possible that T-cells with strong reactivity to AMACR might have tolerance to the antigenic stimulation. Thus, further studies are required to increase the cytotoxic potential of the AMACR-specific CTLs. Moreover, it is reported that AMACR expression is decreased in castration-resistant metastatic diseases [29,30]. In addition, HLA class I expression is decreased in almost 80% of prostate cancer cases as reported by us and other groups [31-33]. The down-regulation of HLA class I was observed more frequently in metastatic sites than in the primary sites. Since HLA class I has a critical role in the recognition of tumor cells by CTLs, defects in antigen presentation could allow the tumor cells to escape from killing by CTLs [34-36]. We showed previously that HLA class I down-regulation was caused at least in part by transcriptional silencing of the β2-microglobulin gene by histone deacetylation in prostate cancer cells, and HLA class I was restored by treatment with histone deacetylase inhibitors [33]. It may be possible for CTL-based vaccines to be used in combination with histone deacetylase inhibitors in immunotherapy for prostate cancer.

## Conclusion

In conclusion, we have provided evidence that AMACR is a potent immunogenic antigen for prostate cancer and AMACR-derived peptides might serve as a cancer vaccine for HLA-A24-positive prostate cancer patients. It is possible that AMACR-targeting therapy might become a rational modality in immunotherapy for various AMACR-expressing cancers.

## Abbreviations

AMACR: alpha-methylacyl-CoA racemase; CTL: cytotoxic T-lymphocyte; PBMC: peripheral blood mononuclear cells; DC: dendritic cell; PHA: phytohemagglutinin; APC: antigen presenting cell.

## Competing interests

The authors declare that they have no competing interests.

## Authors' contributions

IH carried out the CTL induction, killing assays and drafted the manuscript. TT and YH participated in the design of the study and performed the evaluation of the data. TT helped to draft the manuscript. YH contributed to the HLA-A24-binding assay and CTL induction from PBMCs. HK, ES and NM contributed to collecting patients' samples with the informed consent. YT, TT and NS contributed to the design and coordination of this study as well as reviewing the manuscript. All authors have read and approved the final manuscript.
